# Global funding trends for malaria research in sub-Saharan Africa: a systematic analysis

**DOI:** 10.1016/S2214-109X(17)30245-0

**Published:** 2017-06-28

**Authors:** Michael G Head, Sian Goss, Yann Gelister, Victor Alegana, Rebecca J Brown, Stuart C Clarke, Joseph R A Fitchett, Rifat Atun, J Anthony G Scott, Marie-Louise Newell, Sabu S Padmadas, Andrew J Tatem

**Affiliations:** a Global Health Research Institute and Faculty of Medicine, University of Southampton, Southampton, UK; b WorldPop, Department of Geography and Environment, University of Southampton, Southampton, UK; c Department of Social Statistics and Demography and Centre for Global Health, Population, Poverty and Policy, University of Southampton, Southampton, UK; d Southampton NIHR Respiratory Biomedical Research Unit, Southampton General Hospital, Southampton, UK; e Harvard TH Chan School of Public Health, Harvard University, Boston, MA, USA; f Department of Infectious Disease Epidemiology, London School of Hygiene and Tropical Medicine, London, UK; g Flowminder Foundation, Stockholm, Sweden

## Abstract

**Background:**

Total domestic and international funding for malaria is inadequate to achieve WHO global targets in burden reduction by 2030. We describe the trends of investments in malaria-related research in sub-Saharan Africa and compare investment with national disease burden to identify areas of funding strength and potentially neglected populations. We also considered funding for malaria control.

**Methods:**

Research funding data related to malaria for 1997–2013 were sourced from existing datasets, from 13 major public and philanthropic global health funders, and from funding databases. Investments (reported in US$) were considered by geographical area and compared with data on parasite prevalence and populations at risk in sub-Saharan Africa. 45 sub-Saharan African countries were ranked by amount of research funding received.

**Findings:**

We found 333 research awards totalling US$814·4 million. Public health research covered $308·1 million (37·8%) and clinical trials covered $275·2 million (33·8%). Tanzania ($107·8 million [13·2%]), Uganda ($97·9 million [12·0%]), and Kenya ($92·9 million [11·4%]) received the highest sum of research investment and the most research awards. Malawi, Tanzania, and Uganda remained highly ranked after adjusting for national gross domestic product. Countries with a reasonably high malaria burden that received little research investment or funding for malaria control included Central African Republic (ranked 40th) and Sierra Leone (ranked 35th). Congo (Brazzaville) and Guinea had reasonably high malaria mortality, yet Congo (Brazzaville) ranked 38th and Guinea ranked 25th, thus receiving little investment.

**Interpretation:**

Some countries receive reasonably large investments in malaria-related research (Tanzania, Kenya, Uganda), whereas others receive little or no investments (Sierra Leone, Central African Republic). Research investments are typically highest in countries where funding for malaria control is also high. Investment strategies should consider more equitable research and operational investments across countries to include currently neglected and susceptible populations.

**Funding:**

Royal Society of Tropical Medicine and Hygiene and Bill & Melinda Gates Foundation.

## Introduction

The current total domestic and international investments in malaria are considered grossly inadequate to meet the annual global target for investment of US$6 billion.^1^ The 2015 Global Burden of Disease study estimated that there were 731 000 malaria-associated deaths (a decline of about 37% since 2005, along with a decline in age-standardised mortality of 43%)^2^ and 296 million positive cases (a decline of about 30% since 2005) in 2015,^3^ with a high prevalence in sub-Saharan Africa.^4^ To address this burden, malaria is the focus of large and well funded programmes from influential global health actors such as The Global Fund^5^ and the Bill & Melinda Gates Foundation, both of which have targeted malaria for elimination.^6^

An estimated US$8·9 billion was disbursed globally for malaria control and elimination programmes between 2006 and 2010, with most of this funding targeted to Africa.^7^ As well as provision of finance from The Global Fund, substantial investment came from other actors, such as the World Bank and the President's Malaria Initiative. As investment specifically focused on malaria control increases, the burden of malaria decreases,^8^ with interventions estimated to have averted 663 million clinical cases of malaria globally since 2000. Insecticide-treated nets, the most widespread intervention, were responsible for 68% of the averted cases.^9^ However, a substantial burden still remains, requiring efficient allocation of scarce financial resources to address gaps in implementation and research.

The ten largest global health research funders, which include the US National Institutes of Health, the European Commission, the Wellcome Trust, and the Bill & Melinda Gates Foundation, collectively invest about $37·1 billion into research each year,^10^ and malaria is a research priority or part of a wider focus (eg, global health) for these organisations. Investments cover the full pipeline of research, from preclinical science, to clinical trial phases and product development, and on to implementation and operational research. However, few multi-funder analyses of the focus of these awards exist.

**Research in context**

**Evidence before this study**

In July and August, 2016, we searched PubMed and the grey literature (via internet search engines and stakeholder websites, such as WHO) using the search terms “research investments”, “research funding”, “malaria investments”, “malaria funding”, and “malaria Africa” for articles written only in English. One author (MGH) also searched a personal Mendeley literature database that is built for the purpose of informing the Research Investments in Global Health (ResIn) study. Previous investment analyses include the study by Pigott and colleagues, ResIn publications, and the Policy Cures annual reports on product development research in infectious diseases.

**Added value of this study**

To the best of our knowledge, this is the first study to systematically describe the geography of public and philanthropic research funding for malaria in sub-Saharan Africa. The study combined and then re-analysed open data sources from numerous key global health investors, and categorised the awards via the classification system developed by the ResIn study. This strategy allowed us to provide a comprehensive overview of the investment landscape, with actionable data that can help inform equitable decisions around resource allocation.

**Implications of all the available evidence**

The findings show that much of the available resources are directed towards key global health hubs in sub-Saharan Africa—for example, Tanzania, Uganda, and Kenya. However, several countries, such as Chad and Central African Republic, receive little or no research and operational funding despite having high malaria-associated burdens and mortality. These countries have neglected populations, and the global health community should reconsider strategies around resource allocation to reduce inequality and improve equity.

The Research Investments in Global Health study (ResIn) has analysed funding trends in infectious disease research awarded to UK institutions^11^, ^12^ and has identified Africa as being the focus of much of the UK global health research portfolio.^13^ Here, we aimed to systematically analyse investments in research related to malaria from leading international donors, in particular when the focus of the project was in sub-Saharan Africa. We also aimed to locate the site of the research at the national level, describe the geography of investment trends, and compare investments with the local prevalence of malaria caused by *Plasmodium falciparum* and malaria burden, as measured by the sizes of at-risk populations.

## Methods

### Search strategy and selection criteria

The process of collating and categorising infection-related research awards to UK institutions for this systematic analysis has been described in detail elsewhere.^11^, ^12^, ^13^ Briefly, we extracted award data for studies of infectious diseases from funder's websites or requested award data directly from the funder. We also searched funding databases, such as the National Research Register, owned by the UK Department of Health, and clinicaltrials.gov, for infection-related awards. Each award was individually scrutinised and categorised under a number of diseases, disease areas (eg, global health, antimicrobial resistance), and by type of science (eg, phase 1–3 clinical trials, public health research). Award types included project grants, programme grants, fellowships, and pump-priming (development grants) or pilot projects that had a clear research component to the project.

We focused specifically on awards relating to malaria research in sub-Saharan Africa. We used the UK portfolio already collated by the ResIn study^12^ and further considered 28 leading funders of global health research (see appendix 1 for the full list of funders that were assessed, including those that did not have data that met the inclusion criteria). We used existing knowledge and data from the ResIn study, author knowledge, and healthresearchfunders.org to identify key funders who were likely to have provided research investment for malaria. Much of the newly collected data were sourced from the Dimensions for Funders database, UberResearch. When searching online databases for awards related to malaria, we used the search terms “malaria”, “plasmodium”, and “anopheles”. From the retrieved awards, we reviewed the title and abstract to ascertain whether the project had a focus in the 45 sub-Saharan African nations for which data were available. When information about the project was insufficient, we searched databases (including the UK Research Councils' Gateway to Research database, PubMed, and Europe PMC) for publications related to the original award and for information about the award on institutional or study-specific websites. We included awards for which the commitment to fund was dated between 1997 and 2013 (inclusive). The UK Department for International Development and the Bill & Melinda Gates Foundation fund both research and implementation activity; here, we only included the research projects. Using the malaria awards from the preexisting ResIn UK dataset as an example, we included awards of greater than $150 000 (the 10th percentile in the UK dataset). Awards solely related to preclinical science were excluded because they were unlikely to have a specific geographical focus; all other types of science along the research pipeline (from phase 1 studies through to public health and implementation research) were included.

### Data analysis

We categorised the included awards as phase 1–3 trials, intervention and product development (including pharmacovigilance), public health research, or cross-disciplinary research. Public health research included epidemiology, statistics and modelling, and implementation and operational research. Cross-disciplinary research was defined as an award that clearly encompassed two types of science categories (ie, an award covering a phase 3 trial and pharmacovigilance research would be classified as cross-disciplinary). Awards were also categorised by tool or product, specifically diagnostics, vaccines, and therapeutics. Inter-author checks on categorisation yielded a fixed-marginal κ score of 0·88 (indicating a reasonably high level of agreement between authors). All awards were adjusted for 2013 inflation and, when required, were converted to US$ by use of the average exchange rate in the year of the award. South Sudan and north Sudan were unified as Sudan for most of the time period under consideration and were therefore counted together (no investments were specifically for South Sudan after their separation).

Some included awards focused on malaria in multiple sites in multiple countries. Because it was not possible to establish the exact proportion of each award that was invested in or for each site, we used a pragmatic approach to divide the total award size evenly by the number of sites of focus as an approximate measure of likely allocation of resources. For example, for an award of US$1·5 million for malaria research in two sites in Kenya and one site in Tanzania, $1 million would be allocated to Kenya and $500 000 to Tanzania. Similarly, when an award had a clear focus on both malaria and other disease(s), for simplicity of analysis, the total investment was divided by the number of diseases involved. For example, a $1 million award for malaria and tuberculosis in Kenya would have resulted in $500 000 being allocated to malaria research, and the $500 000 assumed to be for tuberculosis research would have been excluded. Awards were not split when the focus was explicitly consideration of malaria as a coinfection with other diseases (the assumption being that all of the funding was for malaria-related burdens).

National-level disbursements of funding for malaria control for 2006–11 were included. Data up to 2010 have been published previously,^7^ and an author (MGH) provided one further year (2011) of unpublished disaggregated information about funding for malaria control (appendix 2). Donors included were national governments, UNICEF, the Global Fund, President's Malaria Initiative, the World Bank, and the Development Assistance Committee. Levels of investment in funding for malaria control and research datasets were compared and ranked at the national level. World Bank data were used to compare 2013 national gross domestic product (GDP).^14^

Data for local prevalence of malaria caused by *P falciparum* were sourced from the Malaria Atlas Project,^15^ which had mapped variables including parasite prevalence and incidence of malaria between 2000 and 2015. We used age-standardised, gridded *P falciparum* parasite prevalence data for ages 2–10 years (P*f*PR_2–10_) at 5 km × 5 km spatial resolution. P*f*PR_2–10_ data were available for all included sub-Saharan African countries, with the exceptions of São Tomé and Príncipe and Comoros. The gridded P*f*PR_2–10_ data were summarised at the country level through integration with WorldPop gridded population data^15^ to construct population-weighted P*f*PR values for each country. All funding data for each country were combined, and maps were produced at the country level to display the total research investment per country between 1997 and 2013.

### Role of the funding source

The funders had no role in the study design, data interpretation, or writing of the report. MGH had full access to all the data in the study and had final responsibility for the decision to submit for publication.

## Results

**Figure 1:**
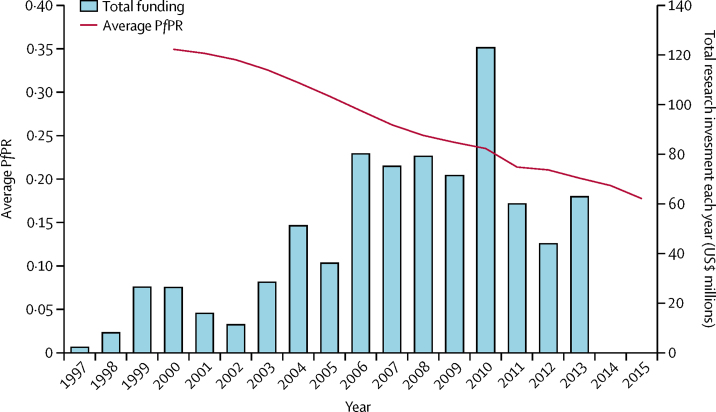
Sum of annual research investment for malaria in sub-Saharan Africa (1997–2013) and mean national-level P*f*PR for all countries in sub-Saharan Africa (2000–15) P*f*PR=*Plasmodium falciparum* parasite prevalence.

By research investment, 333 awards met the inclusion criteria, with a total inflation-adjusted amount for research funding of US$814·4 million (table 1). The mean award size was $2 445 591 (SD 4 054 664) and the median award size was $941 808 (IQR 419 529–$2 605 340). These findings show significant skew in the data, with a small number of very large awards driving the mean higher than the median. The total annual investment for malaria in sub-Saharan Africa varied substantially in the period of 1997–2015, although with a broadly increasing temporal trend alongside observed decreases in disease burden (figure 1). 317 (95%) research awards solely focused on malaria and 16 (5%) awards considered malaria alongside other infections. A total of 285 (86%) awards had just one country of focus.

**Table 1:** Funding for malaria research in sub-Saharan Africa for 1997–2013 by product area and type of science

		**Number of awards**	**Proportion of total awards (%)**	**Total investment (US$)**	**Proportion of total investment (%)**	Mean award size (US$)*	**Median award size (US$)**†
Product area							
	Diagnostics	29	9%	41 010 386	5·0%	14 14 151 (1 670 125)	906 950 (409 574–1 430 935)
	Vaccines	18	5%	64 995 864	8·0%	3 610 881 (3 394 021)	2 909 619 (510 504–4 855 510)
	Therapeutics	83	25%	261 205 837	32·1%	3 147 058 (5 731 588)	991 048 (326 333–3 910 552)
Type of science							
	Phase 1–3 clinical trials	80	24%	275 214 430	33·8%	3 440 180 (4 787 761)	1 317 954 (426 077–4 955 076)
	Intervention and product development	35	11%	87 580 346	10·8%	2 502 296 (4 186 004)	999 485 (293 800–2 605 340)
	Public health	153	46%	308 076 978	37·8%	2 013 575 (4 053 184)	666 164 (375 387–2 209 865)
	Cross-disciplinary	65	20%	143 510 172	17·6%	2 207 849 (2 630 770)	1 071 300 (552 164–2 517 231)
Total		333	NA	814 381 928	NA	2 445 591 (4 054 664)	941 808 (419 529–2 605 340)

NA=not applicable.

* Data are mean (SD).

† Data are median (IQR).

**Figure 2:**
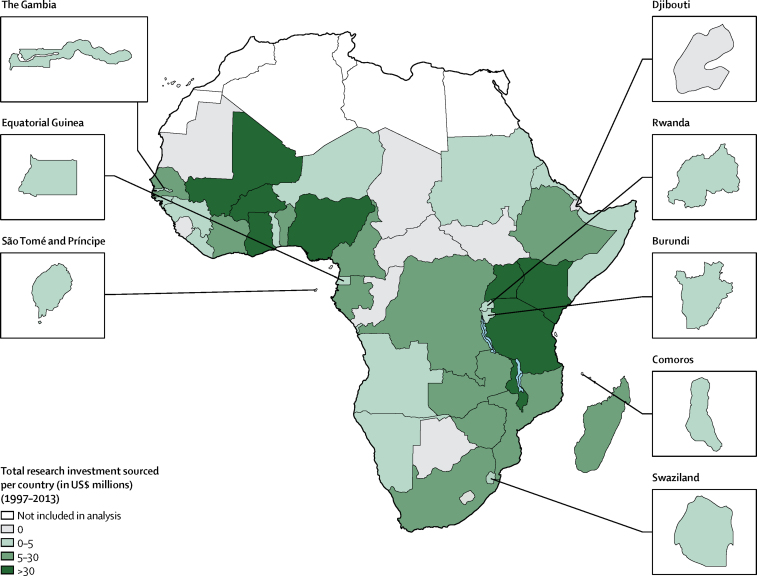
Total research investment (US$ millions) for malaria sourced per country for 1997–2013

**Figure 3:**
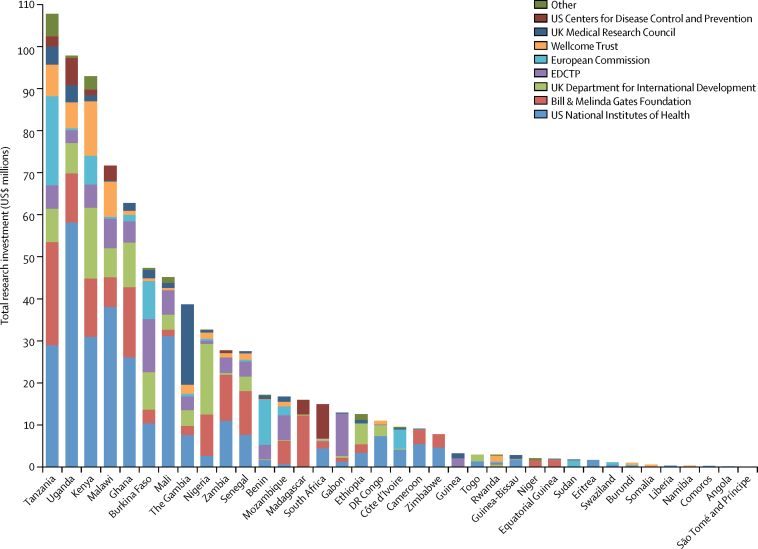
Sum funding for malaria research in sub-Saharan Africa, by country and funder, for 1997–2013 Other included French National Research Agency, National Science Foundation, Research Council of Norway, Swedish Research Council, Swiss National Science Foundation, US Food and Drug Administration, and Economics and Social Research Council. EDCTP=The European & Developing Countries Clinical Trials Partnership. DRC=Democratic Republic of the Congo.

By country, Tanzania, Uganda, Kenya, Malawi, and Ghana were the top five nations to receive the greatest amount of research investment, and 18 nations received greater than US$10 million of research funding (figure 2, figure 3, and table 2). Eight countries were not allocated research investments: Botswana, Cape Verde, Central African Republic, Chad, Congo (Brazzaville), Djibouti, Mauritania, and Sierra Leone.

**Table 2:** Total funding and rankings for malaria research and control investments by country

	**Research investment for malaria**			**Funding for malaria control**		**Combined rankings**		
	Award numbers	Investment allocated (US$)	Ranking	Total funds (US$)	Ranking	Difference between funding and research rankings	Sum of funding and research rankings	Overall ranking*
Tanzania	170	107 769 388	1	749 985 362	2	1	3	1
Kenya	148	92 938 771	3	621 884 255	3	0	6	2
Uganda	115	97 865 177	2	390 362 646	6	4	8	3
Malawi	67	71 664 330	4	424 565 187	5	1	9	4
Nigeria	36	32 639 664	9	786 174 145	1	−8	10	5
Ghana	83	62 725 691	5	345 841 459	8	3	13	6
Mozambique	19	16 358 722	13	350 860 934	7	−6	20	7
Ethiopia	28	12 557 685	17	577 983 173	4	−13	21	8
Zambia	34	27 775 658	10	253 054 196	14	4	24	9=
Madagascar	22	15 944 015	14	309 559 262	10	−4	24	9=
Mali	42	45 135 562	7	175 217 824	18	11	25	11
Senegal	36	27 509 977	11	218 009 244	15	4	26	12
Democratic Republic of the Congo	13	10 930 149	18	331 377 636	9	−9	27	13
Benin	20	17 095 744	12	203 545 688	16	4	28	14=
South Africa	8	14 896 558	15	275 467 512	13	−2	28	14=
Burkina Faso	70	47 333 448	6	106 721 492	23	17	29	16
Rwanda	12	2 933 323	24	299 031 287	12	−12	36	17
The Gambia	61	38 653 641	8	41 275 673	30	22	38	18
Cote d'Ivoire	11	9 535 162	19	129 028 300	20	1	39	19
Togo	4	2 965 449	23	163 947 976	19	−4	42	20
Zimbabwe	6	7 726 559	21	114 130 493	22	1	43	21
Cameroon	15	9 133 108	20	99 854 472	24	4	44	22
Sudan	7	1 749 370	28	178 508 093	17	−11	45	23
Angola	1	117 993	36	300 977 800	11	−25	47	24
Guinea	4	3 224 682	22	44 039 001	28	6	50	25
Gabon	13	12 886 319	16	21 071 247	36	20	52	26
Niger	7	2 122 215	26	64 687 908	27	1	53	27
Liberia	1	297 205	33	121 097 057	21	−12	54	28
Burundi	3	982 238	31	67 946 385	26	−5	57	29
Namibia	4	292 409	34	74 777 578	25	−9	59	30
Equatorial Guinea	4	1 908 866	27	34 065 864	33	6	60	31=
Eritrea	2	1 662 342	29	39 739 873	31	2	60	31=
Guinea-Bissau	5	2 772 862	25	15 118 865	38	13	63	33
Somalia	2	604 537	32	26 459 527	34	2	66	34
Sierra Leone	0	0	38	42 116 404	29	−9	67	35
Swaziland	2	1 049 598	30	12 763 749	40	10	70	36=
Chad	0	0	38	37 921 952	32	−6	70	36=
Congo (Brazzaville)	0	0	38	23 751 153	35	−3	73	38
Mauritania	0	0	38	15 373 467	37	−1	75	39
Comoros	1	224 619	35	7 254 263	42	7	77	40=
Central African Republic	0	0	38	13 812 987	39	1	77	40=
São Tomé and Príncipe	1	27 804	37	7 929 237	41	4	78	42
Djibouti	0	0	38	6 763 868	43	5	81	43
Botswana	0	0	38	5 870 021	44	6	82	44
Cape Verde	0	0	38	2 029 301	45	7	83	45

Countries are ordered by their overall ranking.

* Overall ranking is the sum ranking in order.

By funder, the US National Institutes of Health provided the greatest investment of $292·0 million (36·4%), followed by the Bill & Melinda Gates Foundation, with a total investment of $144·1 million (17·9%); these two funders provided more than 40% of the research investments in all of the top five countries. Some nations were mostly reliant on investment from one funder—for example, the European & Developing Countries Clinical Trials Partnership provided 79% ($10·2 million) of the research investment and was the main funder in Gabon.

**Figure 4:**
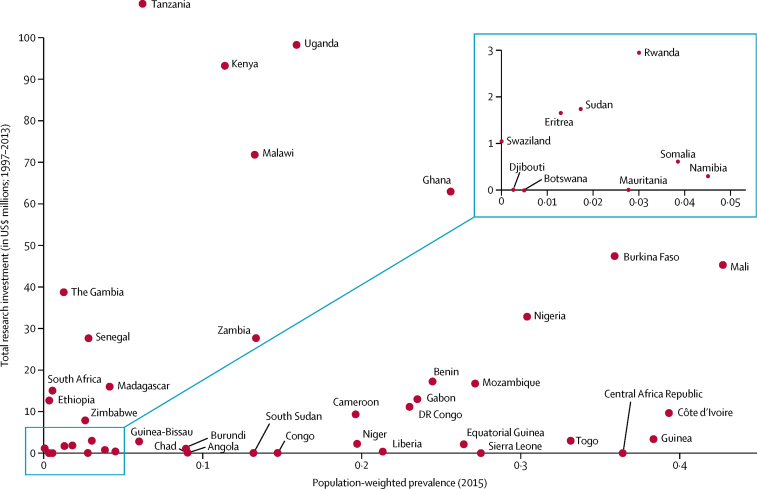
Comparison of 2015 population-weighed parasite prevalence and total research investment received for 1997–2013 by sub-Saharan African nation DRC=Democratic Republic of the Congo.

By type of science (table 1), investment for phase 1–3 clinical trials totalled $275·2 million (33·8%) across 80 (24%) awards. Public health investments totalled $308·1 million (37·8%) across 153 (46%) awards. Cross-disciplinary research covered 65 (20%) awards and product development covered 35 (11%) awards. By product area, 83 (25%) awards were related to research in antimalarial therapeutics, with a total investment of $261·2 million (32·1%). 18 (5%) awards were related to vaccine research ($65·0 million [8·0%]), and 29 (9%) awards focused on diagnostic development ($41·0 million [5·0%]). There was no obvious association between the national population-weighted P*f*PR and the total research investment or funding for malaria control, by country (figure 4; appendix 1). Investments were distributed across high-burden, medium-burden, and elimination settings.

$8·1 billion of funding for malaria control was disbursed in the period of 2006–11 to 45 sub-Saharan African countries (table 2, appendix), with the lowest annual funding in 2006 ($585·2 million, 7·2%) and the highest annual funding in 2010 ($1·9 billion, 23·2%, appendix 2). The top five countries to receive funding in that period were Nigeria ($786·2 million, 9th in malaria research rankings), Tanzania ($750·0 million, ranked 1st in the research rankings), Kenya ($621·9 million, also ranked 3rd in the research rankings), Ethiopia ($578·0 million, ranked 17th in the research rankings), and Malawi ($424·6 million, ranked 4th in the research rankings; table 2). Ghana ranked in the top ten for both level of funding for malaria control (8th) and research investments (5th). Of the eight nations with no allocated research investment, all were ranked in the lowest third (30th or lower out of 45 countries) when considering funding for malaria control, with the exception of Sierra Leone (ranked 29th). The Gambia ranked 30th for funding for malaria control, but 8th for research investments. Most nations ranked similarly for both research investments and funding for malaria control.

Combined rankings showed the top five nations to be Tanzania, Kenya, Uganda, Malawi, and Nigeria. When adjusting for GDP (appendix), the top five nations to rank in the top ten across both funding for malaria control and research investment were Malawi, Tanzania, Uganda, Kenya, and Madagascar.

## Discussion

Many research investments for malaria were focused in sub-Saharan Africa. We found that the greatest investments were allocated to Tanzania, Uganda, and Kenya. Malawi, Ghana, and Nigeria also ranked highly across both research investment and funding for malaria control. Investments for research were typically highest in countries where funding for malaria control was also high (The Gambia and Nigeria being notable exceptions). Similarly, most nations receiving little or no research investment for malaria also received scarce funding for malaria control, despite typically having a reasonably high malaria-related burden of disease. About a third of research investments were related to antimalarial therapeutics. The US National Institutes of Health and the Bill & Melinda Gates Foundation provided about 60% of research funding for malaria in sub-Saharan Africa, and funding was disbursed to countries with high and low burdens of malarial disease, indicating that drivers for allocation of investments are likely to include the desire to both reduce existing high burdens and to pursue efforts towards elimination in relatively low-burden settings. There appeared to be inequalities in overall disbursement of funding for malaria research, which will probably help to accelerate elimination in some settings but might neglect other vulnerable populations in locations of high malarial burden. Investments in malaria control broadly reflected the trends in research investment.

Evidence that investments in malaria research contribute to improved health outcomes is scarce, in part because of the numerous and complex variables that must be considered when assessing resource allocation. Thus, the correlation between increased research investment and decreased burden cannot in itself be considered causal. Funding for malaria control also increased between 2006 and 2011, and interventions, including bednets and therapeutics, have clearly had substantial impacts.^9^ Case studies provide qualitative evidence to justify investments in global health research,^16^ and the findings presented here lend some weight to that conclusion. To qualify and address the gaps in evidence, WHO has established the Global Observatory on Health Research and Development,^17^ and is openly seeking evidence-based contributions to assimilate existing knowledge and inform global strategies.^18^ The ResIn study is also scheduled to report findings of a global research investments analysis for all infectious diseases in 2017.

Investing requires confidence on the part of the investor that they will see a return on their investment (for example, health gains from operational investments such as provision of insecticide-treated nets, or research investments that generate new knowledge or products such as vaccines and diagnostics). Therefore, particularly in environments where the logistics for research might be complex and challenging, the inclination is to fund governments and institutions with a track record of success and in locations where it is perceived that the investment will make a positive difference and where any research will be feasible. Thus, countries such as Tanzania, Uganda, and Kenya, which all have existing relatively good infrastructures for research (such as the Kenya Medical Research Institute sites in Nairobi and Kilifi), continue to receive steady sums of research investment and also benefit from relatively high levels of funding for malaria control. From these streams of funding, high-quality research that provides clarity on the research questions under investigation is likely. Conversely, funders do not have the incentive or confidence to invest in African nations such as Chad, Somalia, and the Central African Republic, which are considered politically fragile and have inadequate infrastructure and regulations surrounding business start-ups and trading.^19^ The USA and the UK are leading investors in global health and, specifically, in provision of funding for malaria, but the changeable political climates in both countries might have implications for global health investment and activity.

Other factors that influence resource allocation include political and socioeconomic factors such as conflict, corruption, and crime and economic considerations. Poorer nations with weak infrastructures are less likely to reap the benefits from research investment without improvements in health systems. For example, eight of the top ten nations considered most susceptible to infectious disease outbreaks are in sub-Saharan Africa, and the three most susceptible are Somalia (ranking 34th in our investment rankings), Central African Republic (ranking 40th in our investment rankings), and Chad (ranking 36th in our investment rankings).^20^ Corruption indices rank Somalia as the worst performer, with numerous sub-Saharan African nations ranked in the lower percentiles.^21^ In a systematic analysis^22^ of the geographical distribution of global health partnerships with the 100 highest-ranked universities worldwide, Kenya, Tanzania, and Uganda had 43 partnerships. Of the 12 countries that ranked lowest in our investment rankings, only Sierra Leone, with five partnerships, and Botswana, with three partnerships, had any recorded global health partnerships.^22^ The global health partnership analysis^22^ also described areas in northern and central Africa that had either few or no partnerships with high-ranking universities, despite these areas being in great need of health care and thus surely also in particular need of investment (institutions ranked outside of the top 100 will potentially have international partnerships that were undocumented in the global health partnership analysis and might include the lower-ranked nations here). Capacity building and training initiatives, and geographical priorities set by research funders, might help incentivise leading academic institutions to develop new partnerships with sub-Saharan African countries. Our findings correspond with other analyses^23^ in showing broadly low sum investments in central Africa, with resources mostly concentrated in western and eastern Africa. High malaria mortality and low coverage (<50%) of insecticide-treated bednets are seen in lower-ranked countries in these central regions, such as Congo (Brazzaville), Guinea, and Central African Republic.^14^ The five countries ranked lowest in terms of their likelihood to meet the targets set in the health-related UN Sustainable Development Goals were Central African Republic, Somalia, South Sudan, Niger, and Chad.^24^ There are huge differences in the amount of investment each high-income nation will allocate to global health operational and research investments and so, when the amount of resource allocated to each low-income or middle-income nation is linked to the wealthier nation's historical ties,^13^ there is the potential for inequalities in resource allocation to be introduced and for health issues to be inadequately addressed.

Research outputs can translate across national borders, but might not do so without appropriate consideration of the context in which research findings are used. Substantial advantages exist in investment in local research, particularly with regards to ownership of the results, trust, inter-sector sharing of expertise between researchers and policy makers, and increased contextualisation of findings.^25^ If little or no local track record in research exists, then inter-sector expertise might be less likely.^26^ Decision-makers will assess research findings by taking into account the political context,^27^ the capacity of local health systems to absorb the knowledge and put the innovation into practice, and the appropriateness of the proposed intervention.^28^ *The Lancet's* Commission on Global Health 2035^29^ highlighted how low-income nations such as Chad or Somalia could experience substantial health gains with an “enhanced R&D investment scenario” deployed at a national level, alongside societal benefits. Investment in biomedical science research and development in the UK yields a 17% return to the economy, and each pound of public investment stimulates the same investment from the private sector.^30^ New investment in research and development in low-income settings could potentially be the catalyst for improved health systems and confidence in the business and academic sectors.

In 2015, WHO announced a new global strategy for addressing malaria between 2016 and 2030, for which the target is to reduce the global malaria burden by 90% before 2030.^4^ Innovation and research is one of the three major pillars of that strategy. WHO estimates that additional funding of $673 million annually is needed for malaria research,^4^ a substantial increase compared with current funding, and that $6·4 billion is needed every year for funding of malaria control.^1^ Research recommendations include analysis of transmission-blocking medicines in high-transmission settings, investigation of the short-term and long-term effects of administration of effective antimalarials, implementation research that refines approaches to application of existing interventions to make them more effective and more efficient in local contexts, diagnostics that detect low-level parasitaemia in asymptomatic carriers, and research to develop more effective vaccines. The African Union health strategy for years 2016–30 aims to “increase investments in health, improve equity and address social determinants of health”.^31^ One of the strategic areas is to “end malaria”,^31^ although little specific detail is available on how this could be achieved. Further tracking of resource allocation, such as with research portfolios, is essential to inform strategies that will allow WHO's ambitious 90% reduction target to be met.

Limitations of the ResIn analyses have been previously described in full.^11^, ^12^ The research funding data included here were sourced from probably the largest public and philanthropic contributors to global health and malaria-related research; however, resource constraints prevented inclusion and analysis of other national funders who might provide further investment in their countries of focus. We also excluded preclinical science and malaria research focused on nations outside of sub-Saharan Africa, although findings from such studies might eventually inform sub-Saharan-African-focused strategies. The Global Fund and President's Malaria Initiative typically do not consider their investments as research but as operational activity; thus, they were not included in the research analyses but were included in the funding for malaria control dataset.^7^ The Bill & Melinda Gates Foundation provides investments for both research and operational activity; however, scarce information was openly available on the Foundation's grants database, and categorisation of these awards was therefore sometimes difficult. Private sector investments were not available for detailed systematic analyses and thus were not included, leading to a clear data gap, particularly in investment directed towards research for new products. However, almost all financing for malaria vaccine research and drug development comes from public and philanthropic sources.^32^ Some of the research investments not included here in the public, philanthropic, or private sectors might have been focused on countries that we found to be neglected in terms of receipt of investment in our analysis. Although we found correlations between temporal levels of investment and disease burden, causality could not be inferred from those results. The categorisation process was subjective and we made assumptions about allocations of awards, athough we took care to reduce subjectivity; the high κ score on inter-observer concordance indicates a high level of agreement between authors and inter-author checks reduced the likelihood of systematic error.

To the best of our knowledge, this is the first study to systematically analyse both research and operational investments for malaria in sub-Saharan Africa. Reasonable consistency in amounts of FMC and research investment was seen in those African nations that benefit from receipt of significant shares of available resources and consistent inequalities were seen in those that do not; many countries receiving little in the way of investment are those with the highest burden of malaria and the weakest health systems and infrastructures. Evidence suggests that ownership of research findings increases their uptake and that, although doing research in some settings is extremely challenging because of negative political and socioeconomic factors, these areas contain some of the world's most vulnerable populations. Thus, careful investment strategies should consider how these populations can best be reached.
