# Supplementary material for: Global funding trends for malaria research in sub-Saharan Africa: a systematic analysis
Source: Lancet Glob Health. 2017 Jun 28;5(8):e772–81. doi: 10.1016/S2214-109X(17)30245-0 (PMC5567191; doi:10.1016/S2214-109X(17)30245-0)
Supplement: Supplementary Appendix 1 [file mmc1.pdf]

THE LANCET
Global Health

Supplementary appendix

This appendix formed part of the original submission and has been peer reviewed. We post it as supplied by the authors.

Supplement to: Head MG, Goss S, Gelister Y, et al. Global funding trends for malaria research in sub-Saharan Africa: a systematic analysis. *Lancet Glob Health* 2017; published online June 28. http://dx.doi.org/10.1016/S2214-109X(17)30245-0.

Supplementary information

Supplementary I – Funding for malaria control data from Pigott et al (see spreadsheet included with manuscript)

Supplementary II – Funders considered for this study

Supplementary III – R&D and FMC investment and national GDP

Supplementary IV – Funding for malaria control 2006-2011, by country

Supplementary V – Parasite prevalence and funding for malaria control 2006-2011, by country

Funders

**Included funder**	**Source**	**Complementary source**
US Centre for Disease Prevention and Control (CDC)	Funder Website	-
UK Department for International Development (DFID)	Funder Website	-
European Commission	Spreadsheets emailed directly from EC	Resin spreadsheet (previously collated data)
European & Developing Countries Clinical Trials Partnership (EDCTP)	Funder website	
Bill & Melinda Gates Foundation	Funder Website	Resin spreadsheet (previously collated data)
INSERM	Funder Website	-
Institut Pasteur	Funder Website	-
UK Medical Research Council	Resin spreadsheet (previously collated data)	Dimensions
US National Institute for Health	Dimensions	-
Research Council of Norway	Dimensions	-
Swedish Research Council	Dimensions	-
Swiss National Science Foundation	Dimensions	-
Wellcome Trust	Resin spreadsheet (previously collated data)	Dimensions
**Funders checked but not included**	**Reason for exclusion**	
Canadian Institutes of Health Research	No data meeting inclusion criteria	
Centre National de la Recherche Scientifique	No adequate or identifiable database	
Chinese Ministry of Health	No adequate or identifiable database	
German Federal Ministry of Education and Research	No adequate or identifiable database	
German Research Foundation	No adequate or identifiable database	
Howard Hughes Medical Institute	No data meeting inclusion criteria	
Indian Council of Medical Research (ICMR)	No adequate or identifiable database	
Innovative Medicines Initiative	No adequate or identifiable database	
Instituto de Salud Carlos III	No adequate or identifiable database	
Japan Science and Technology Agency	No adequate or identifiable database	
Japan Society for Promotion of Science	No adequate or identifiable database	
Medicines for Malaria Venture	No adequate or identifiable database	
Ministry of Health of Italy	No adequate or identifiable database	
National Health & Medical Research Council (Australia)	No data meeting inclusion criteria	
National Natural Science Foundation of China	No adequate or identifiable database	
Singapore National Medical Research Council	No adequate or identifiable database	

Country	GDP per capita (US$)	FMC per GDP (US$)	Ranking FMC	R&D per GDP (US$)	Ranking R&D	Combined ranking score	Overall ranking
Malawi	341	1245500	1	210234	1	2	1
Tanzania	909	824767	3	118515	3	6	2
Uganda	674	578880	7	145127	2	9	3
Kenya	1261	493131	8	73697	5	13	4=
Madagascar	463	668632	5	34438	8	13	4=
Democratic Republic of Congo	414	801018	4	26421	11	15	6=
Ethiopia	504	1147014	2	24921	13	15	6=
Mozambique	605	579711	6	27029	10	16	8
Mali	798	219484	15	56538	7	22	9
Burkina Faso	709	150509	20	66754	6	26	10=
Ghana	1827	189284	17	34331	9	26	10=
The Gambia	484	85260	24	79844	4	28	12=
Senegal	1051	207356	16	26166	12	28	12=
Benin	883	230610	14	19369	14	28	12=
Nigeria	2980	263831	12	10953	16	28	12=
Togo	589	278343	10	5035	22	32	16
Rwanda	679	440401	9	4320	24	33	17
Zambia	1840	137565	21	15099	15	36	18
Burundi	259	261972	13	3787	25	38	19
Zimbabwe	906	126041	22	8533	17	39	20=
Niger	418	154878	18	5081	21	39	20=
Cote d'Ivoire	1447	89156	23	6589	19	42	22=
Liberia	453	267122	11	656	31	42	22=
Cameroon	1331	75011	26	6861	18	44	24
Guinea	522	84440	25	6183	20	45	25
Sudan	1157	154220	19	1511	28	47	26
Eritrea	543	73186	27	3061	26	53	27
Guinea-Bissau	584	25876	34	4746	23	57	28
South Africa	6882	40028	32	2165	27	59	29

Somalia	521	50764	30	1160	30	60	30
Angola	5327	56499	28	22	36	64	31
Sierra Leone	803	52479	29	0	38	67	32
Central African Republic	317	43551	31	0	38	69	33
Comoros	797	9105	37	282	33	70	34=
Namibia	5421	13795	35	54	35	70	34=
Gabon	10659	1977	42	1209	29	71	36=
Chad	985	38496	33	0	38	71	36=
Swaziland	3648	3499	41	288	32	73	38
Mauritania	1458	10545	36	0	38	74	39
Sao Tome and Principe	1676	4732	39	17	37	76	40=
Congo	3205	7410	38	0	38	76	40=
Equatorial Guinea	21498	1585	43	89	34	77	42
Djibouti	1683	4018	40	0	38	78	43
Botswana	6807	862	44	0	38	82	44
Cape Verde	3623	560	45	0	38	83	45

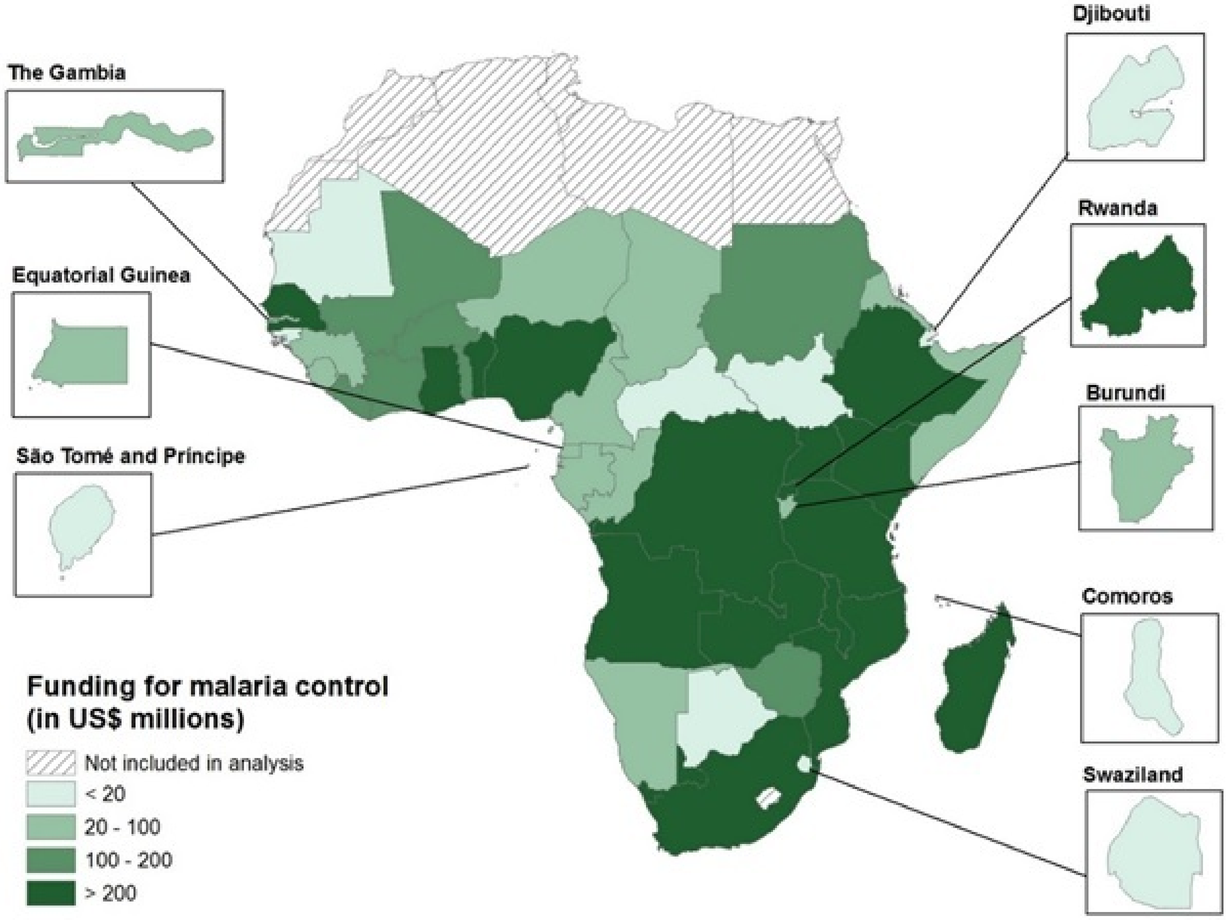
The Gambia
Equatorial Guinea
São Tomé and Príncipe
Djibouti
Rwanda
Burundi
Comoros
Swaziland
Funding for malaria control
(in US$ millions)
Not included in analysis
< 20
20 - 100
100 - 200
> 200

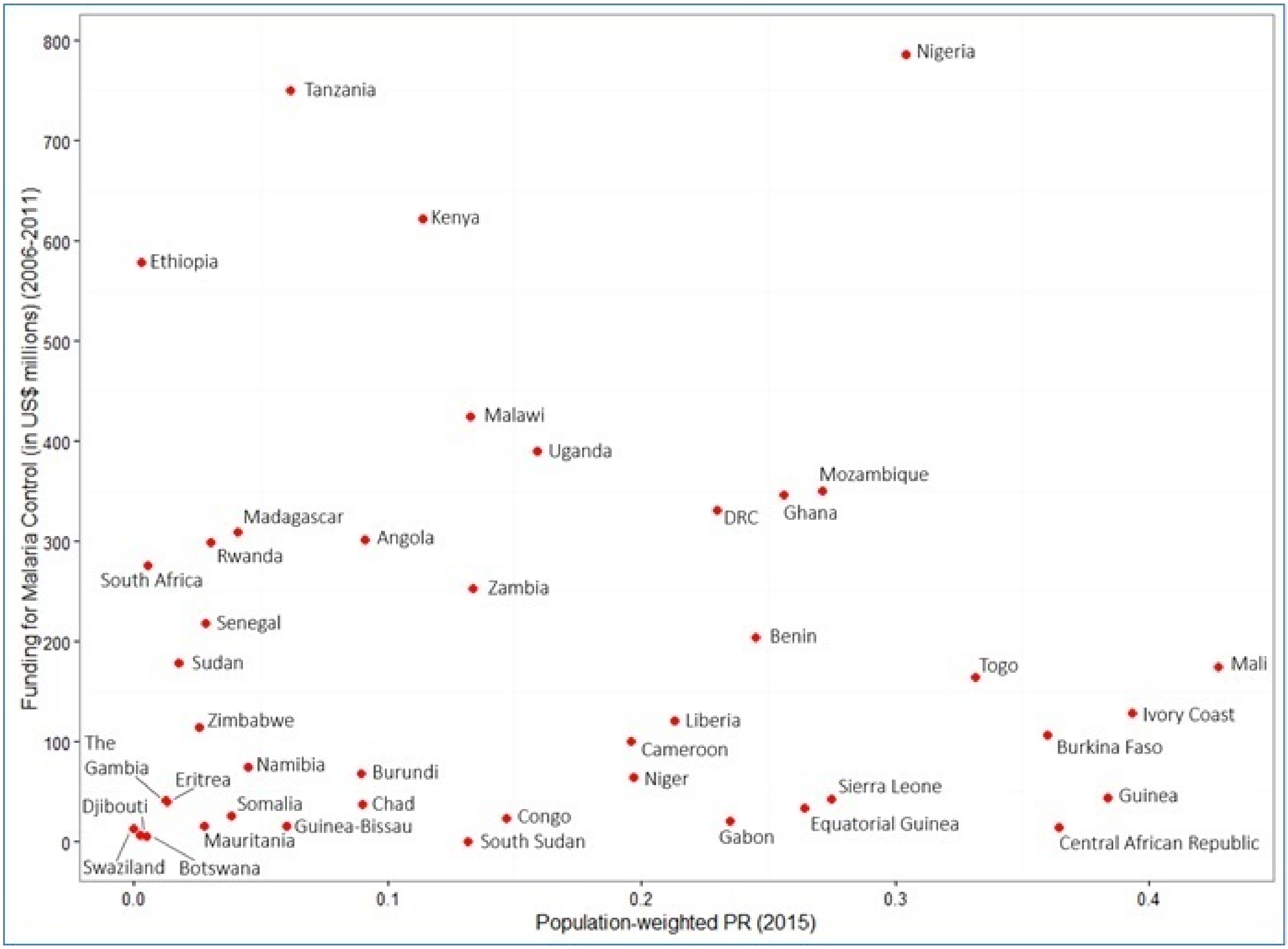

Funding for Malaria Control (in US$ millions) (2006-2011)
Population-weighted PR (2015)
0
100
200
300
400
500
600
700
800
0.0
0.1
0.2
0.3
0.4
Nigeria
Tanzania
Kenya
Ethiopia
Malawi
Uganda
Mozambique
Ghana
DRC
Madagascar
Angola
Rwanda
South Africa
Zambia
Senegal
Benin
Sudan
Togo
Mali
Zimbabwe
Liberia
Ivory Coast
Burkina Faso
Cameroon
The Gambia
Eritrea
Namibia
Burundi
Niger
Sierra Leone
Guinea
Djibouti
Somalia
Chad
Congo
Equatorial Guinea
Gabon
Mauritania
Guinea-Bissau
South Sudan
Central African Republic
Swaziland
Botswana
